# Individual Baseline Performance and Electrode Montage Impact on the Effects of Anodal tDCS Over the Left Dorsolateral Prefrontal Cortex

**DOI:** 10.3389/fnhum.2020.00349

**Published:** 2020-09-08

**Authors:** Maike Splittgerber, Ricardo Salvador, Hannah Brauer, Carolin Breitling-Ziegler, Alexander Prehn-Kristensen, Kerstin Krauel, Rafal Nowak, Giulio Ruffini, Vera Moliadze, Michael Siniatchkin

**Affiliations:** ^1^Institute of Medical Psychology and Medical Sociology, University Medical Center-Schleswig Holstein, Kiel University, Kiel, Germany; ^2^Neuroelectrics Barcelona, Barcelona, Spain; ^3^Department of Child and Adolescent Psychiatry, Center for Integrative Psychiatry Kiel, University Medical Center Schleswig-Holstein, Kiel, Germany; ^4^Department of Child and Adolescent Psychiatry and Psychotherapy, Otto-von-Guericke University Magdeburg, Magdeburg, Germany; ^5^Center for Behavioral Brain Sciences (CBBS), Magdeburg, Germany; ^6^Clinic for Child and Adolescent Psychiatry and Psychotherapy, Medical Center Bethel, Bielefeld, Germany

**Keywords:** tDCS, working memory, montage, individual performance, DLPFC

## Abstract

Anodal transcranial direct current stimulation (tDCS), applied over the left dorsolateral prefrontal cortex (lDLPFC), can produce significant effects on working memory (WM) performance and associated neurophysiological activity. However, results from previous studies are inconsistent and occasionally contradictory. This inconsistency may be attributed to methodological and individual differences during experiments. This study therefore investigated two hypotheses: (1) A multichannel-optimized montage was expected to be more effective than a classical bipolar montage, because of increased focality. (2) The subjects were expected to benefit differently from the stimulation depending on their initial task performance. In a sham-controlled crossover study, 24 healthy participants received bipolar, multichannel, and sham stimulation for 20 min in randomized order, targeting the lDLPFC while performing a 2-back WM task. After stimulation, electroencephalography (EEG) was recorded at rest and during 2-back and nontarget continuous performance task (CPT) performance. Bipolar and multichannel stimulations were both well tolerated and effectively blinded. We found no effect of stimulation on behavioral performance or neuronal oscillations comparing the classical bipolar or multichannel montage with sham stimulation. We did, however, find an interaction between stimulation and initial task performance. For multichannel stimulation, initially low-performing participants tended to improve their WM performance while initially high-performing participants tended to worsen their performance compared to sham stimulation. Both tDCS montages induced changes in neural oscillatory power, which correlated with baseline performance. The worse the participants’ initial WM performance was, the more task-related theta power was induced by multichannel and bipolar stimulation. The same effect was observed for alpha power in the nontarget task following multichannel stimulation. Notably, we were not able to show a superiority of multichannel stimulation compared to bipolar stimulation. Still, comparing both montages with sham stimulation, multichannel stimulation led to stronger effects than bipolar stimulation. The current study highlights the importance of investigating different parameters with potential influence on tDCS effects in combination. Our results demonstrate how individual differences in cognitive performance and electrode montages influence effects of tDCS on neuropsychological performance. These findings support the idea of an individualized and optimized stimulation setting, potentially leading to increased tDCS effects.

## Introduction

Working memory (WM) is a cognitive function that underlies a multitude of our daily activities and is central to our thoughts and actions. It describes the ability to maintain information for a brief time interval in an active and easily accessible state (Baddeley and Della Sala, 1996; Kane and Engle, 2002; Baddeley, 2010, 2012; Chai et al., 2018). A variety of mental disorders, such as schizophrenia (Galderisi et al., 2009) or attention-deficit/hyperactivity disorder (ADHD; Brennan and Arnsten, 2008), are associated with WM impairments. Improvement of WM may increase the adaptability of affected individuals and their quality of life. However, most WM trainings have been characterized by a limited generalization and low-enduring effects (Redick et al., 2013; Soveri et al., 2017).

Transcranial direct current stimulation (tDCS) appears to provide a method to enhance the effectiveness of WM trainings. tDCS is a noninvasive brain stimulation technique that induces changes in cortical excitability through the modulation of the membrane potential in cortical neurons (Nitsche and Paulus, 2000) that can last beyond the duration of the stimulation (Nitsche and Paulus, 2001; Hoy et al., 2013; Paulus et al., 2016). Studies using electroencephalography (EEG) demonstrated that tDCS can alter brain activity in different target areas and related networks (Zaehle et al., 2011; Miller et al., 2015; Bergmann et al., 2016; Wörsching et al., 2016; Jones et al., 2017). The left dorsolateral prefrontal cortex (lDLPFC) is a brain region strongly associated with WM processes (D’Esposito et al., 1995; Mansouri et al., 2009). A variety of studies have illustrated improved WM performance during or after tDCS over the lDLPFC (Fregni et al., 2005; Hoy et al., 2013; for review see Brunoni and Vanderhasselt, 2014; Dedoncker et al., 2016; Hill et al., 2016). However, various studies have failed to show improved WM performance (Brunoni and Vanderhasselt, 2014; Hill et al., 2016; Dumont et al., 2018; Röhner et al., 2018) or cortical reactivity (Boonstra et al., 2016; Gordon et al., 2018; Hill et al., 2018) caused by lDLPFC stimulation. Based on these results, it seems necessary to identify and investigate factors that have an influence on tDCS-induced effects on WM.

One potential factor influencing tDCS effects is the electrode montage, which affects the current flow or, equivalently, electric-field (*E*-field) distribution. Most studies targeting the lDLPFC use a bipolar montage with the anode placed over F3 and the cathode placed over the supraorbital region, corresponding to the international 10-20 system (Herwig et al., 2003). This montage leads to a rather diffuse *E-field* distribution and therefore poor spatial targeting, according to computation modeling studies (Miranda et al., 2013; Saturnino et al., 2015; Laakso et al., 2016). One promising way of achieving more focal stimulation is through multichannel optimized montages, using several small electrodes distributed on the head. Recently, optimized multichannel tDCS over the motor cortex has been shown to increase motor cortex excitability, with significantly greater effects than bipolar tDCS (Fischer et al., 2017). Additionally, several studies recently investigated a ring-shaped 4 × 1 high-definition-tDCS (HD-tDCS) targeting the lDLPFC, showing increased effects on neurophysiological activity and WM performance (Nikolin et al., 2015; Hill et al., 2017, 2018, 2019). However, there is no study investigating multichannel tDCS over the lDLPFC using an optimized distributed electrode montage rather than a ring-shaped HD-tDCS montage.

Another factor that can potentially explain differences in tDCS effects on WM is the interindividual variability in participant baseline WM performance. Studies investigating the effect of tDCS on WM performance and related neural activity report inconsistent effects on initially high and low performers (Jones and Berryhill, 2012; Tseng et al., 2012; Gözenman and Berryhill, 2016; Hsu et al., 2016). Furthermore, different studies report a negative linear relationship between initial baseline performance and tDCS effects for different modalities. The worse the subjects initially perform, the more likely they are to benefit from the stimulation (Rosen et al., 2016; Habich et al., 2017). Despite their contradictory results, these studies underline the potential predictive power of interindividual WM capacity on tDCS outcome.

Based on these findings, in our study we combined both factors (electrode montages and individual baseline performance) as possible predictors for effects on behavioral and neurophysiological outcomes induced by tDCS over the lDLPFC. We included a 2-back WM task as target task and a continuous performance task (CPT) as nontarget task to test for nonspecific tDCS effects and to differentiate stimulation effects more clearly. The CPT investigates response-inhibition and attention (Rosvold et al., 1956), functions that are also connected to the DLPFC (Blasi et al., 2006; Oldrati et al., 2016). We expected: (1) an improvement in WM performance during and after bipolar and multichannel tDCS compared to sham, with pronounced effects for multichannel stimulation compared to bipolar stimulation. (2) We expect that these modifications in WM performance should be reflected in changes in neural oscillatory power during task processing. Regarding the predictive role of baseline performance on tDCS outcome, we expected that individual baseline performance predicts; (3) changes in behavioral performance; and (4) changes of oscillatory power induced by tDCS.

## Materials and Methods

### Participants

The study was approved by the Ethics Committee of the Faculty of Medicine Kiel, University Kiel. All participants gave their written and informed consent prior to the start of the experiment. To calculate sample size, we used G*Power (Faul et al., 2007) with the following settings: effect size *f* = 0.25 following Brunoni and Vanderhasselt (2014) and Dedoncker et al. (2016), α level = 0.05, power = 0.95, correlation among repeated measures = 0.7. The minimum sample size was found to be 22. To fully counterbalance the order of stimulation conditions across participants, we included 24 subjects (mean age 24.8, SD = 2.7 years; 13 females). Exclusion criteria were relevant psychological problems, assessed by the SCL-90-R (Franke, 2002), ADHD-related symptoms assessed by the ADHS-E (Schmidt and Petermann, 2009), depression-related symptoms assessed by the beck depressions-inventar (BDI-II; Hautzinger et al., 2006), IQ score below 85, evaluated by the CFT 20-R (Weiß, 2006), history of neurological or psychiatric diseases, use of medication, pregnancy, or metallic-head implants (see Table 1). All subjects were naive to transcranial stimulation. Furthermore, besides the general information given in the consent, all subjects were naïve with regard to the aim of the study. Subjects received money or research credits for their participation.

**Table 1:** Subject characteristics.

	Mean ± standard deviation	Exclusion criteria
Sex	13 females, 11 males
Age (years)	24.83 ± 2.72	18 < age > 30
BDI II total score	4.21 ± 3.78	BDI > 13
ADHS-E T-value main scale	47.92 ± 8.67	*T* > 60
SCL-90-R T-value GSI	47.5 ± 9.23	*T* > 65
CFT-20-R	113.13 ± 12.72	IQ < 85

### Experimental Design

**Figure 1:**
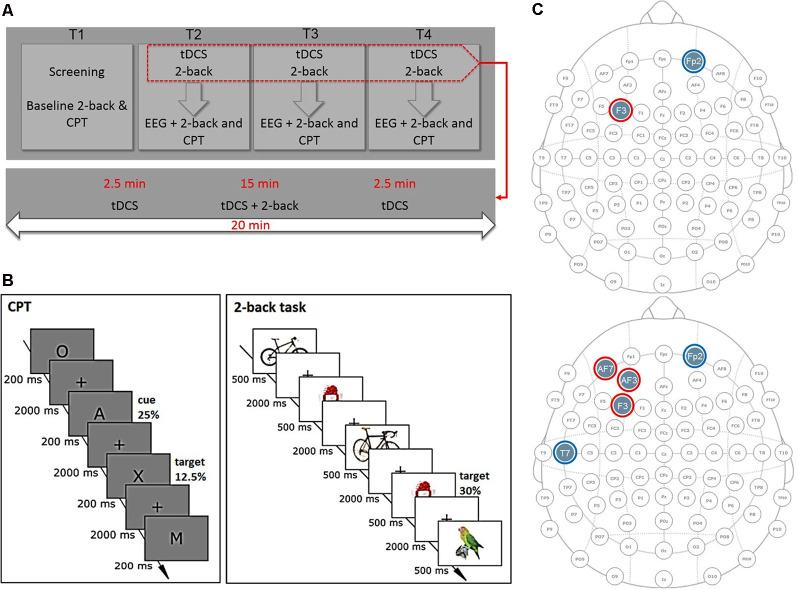
Experimental design. **(A)** Time-course of the experiment. Each participant attended four sessions. At screening and baseline sessions (T1), we assessed inclusion and exclusion criteria and task baseline performance. At stimulation sessions (T2–T4), participants were stimulated for 20 min with consecutive 2-back task performance. After stimulation, electroencephalography (EEG) at rest and during task performance was recorded. **(B)** Tasks. In the 2-back task, participants had to decide whether a currently presented picture was identical to the picture shown two steps back. In the continuous performance task (CPT), participants had to press the space bar every time the letter A was followed by the target letter “X” and withhold their response for all other letters. **(C)** Electrode montages for bipolar stimulation (top) and multichannel stimulation (bottom). Red circles represent anodal; blue circles reference electrodes.

We used a randomized, sham-controlled, single-blind, crossover design. All participants attended four sessions: one screening and baseline measurement (T1) followed by three stimulation sessions (T2–T4; Figure 1A). The order of stimulation sessions (sham, multichannel, and bipolar montage) was randomized and balanced across participants. The period between sessions for a single subject was a minimum of 7 days and a maximum of 11 days. In 90% of the cases a time interval of 7 days was kept. Each stimulation session started with a 20-min stimulation. After 2.5 min of stimulation, the 2-back task started and ended 2.5 min before the end of stimulation to prevent distraction induced by current ramping and to allow related side effects to wear off. After the stimulation, participants filled in a questionnaire on safety, tolerability, and blinding of stimulation. Subsequently, a 64-channel EEG was then set up within 45 min and EEG during rest with eyes closed and opened (2 × 2 min) and during 2-back and CPT performance was recorded.

### Tasks and Stimuli

Both tasks were programmed using the software Presentation^®^ (Version 20.0, Neurobehavioral Systems, Inc., Berkeley, CA, USA^1^). Prior to the start of each task, participants completed a training session and were instructed to react as fast and accurately as possible. The investigator made sure the tasks were fully understood.

In the 2-back task, participants had to decide whether a currently presented picture was identical to the picture shown two steps back (Figure 1B). The 2-back task lasted approximately 15 min and consisted of 360 trials with 30% target trials. Participants had to press the right mouse button if the trial was a nontarget or the left mouse button if the trial was a target trial. Each trial consisted of a 500-ms picture presentation followed by a fixation cross presentation jittered between 1,550 and 2,000 ms duration, resulting in a trial duration of 2,050–2,500 ms. We used seven sets of 16 different pictures taken from the Mnemonic Similarity Task (MST), Stark lab^2^, as stimuli, one for the baseline and screening sessions, two for each stimulation session.

In the CPT, various uppercase letters were presented (Figure 1B). Participants had to press the space bar every time a target letter was presented (letter “X”) and withhold their response for all other letters. The target was always preceded by a cue stimulus (letter “A”), whereby the cue could be followed by a target or nontarget letter. The CPT lasted approximately 18 min and consisted of 480 trials with 25% cue and 12.5% target letters. Each stimulus was presented for 200 ms followed by a fixation cross for 2,000 ms, leading to a trial duration of 2,200 ms.

### Transcranial Direct Current Stimulation

Participants were stimulated three times with either bipolar, multichannel, or sham stimulation over the lDLPFC for 20 min using the Starstim 32 stimulator (Neuroelectrics, Barcelona, Spain). Electrodes were positioned using a head cap following the 10-10 system (Figure 1C). For bipolar stimulation, 1 mA tDCS was delivered by a pair of circular saline-soaked surface sponge electrodes (25 cm^2^), with the anode positioned over F3 and cathode over Fp2. For multichannel tDCS, we used five 3.14-cm^2^ circular PiStim electrodes, positioned at AF3 (897 μA), AF7 (284 μA), F3 (819 μA), Fp2 (−1,000 μA), and T7 (−1,000 μA), filled with EEG electrode gel. In both conditions, current was ramped up for 30 s at the beginning and down during 30 s at the end of stimulation. In the sham condition, half of the subjects received a multichannel, the other half a bipolar montage. Current was ramped up and immediately down for 60 s at the beginning and end of the stimulation.

#### Computational Modeling of Electric Fields

**Figure 2:**
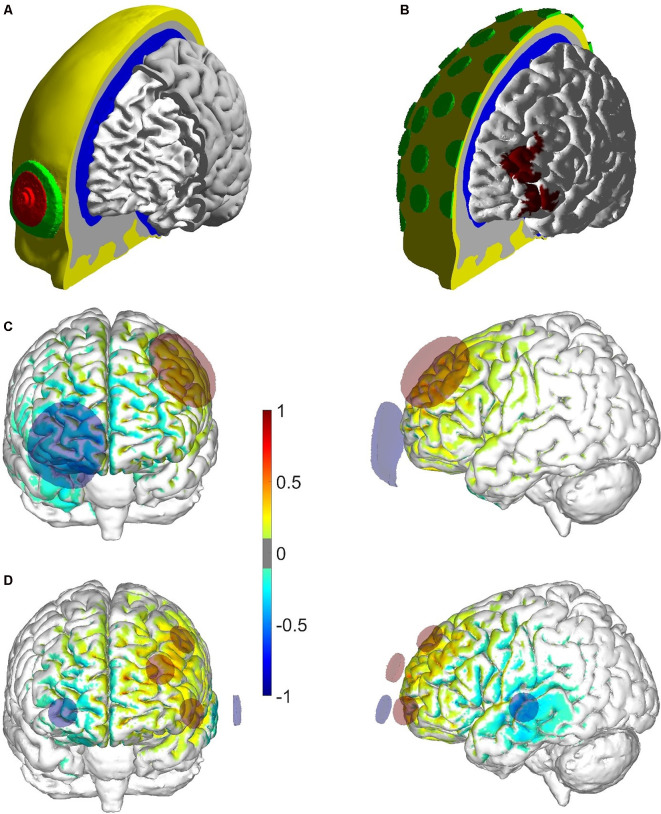
Geometry of the head model used in the optimization and *E*-field (E*_n_*) modeling pipeline. **(A)** Global view of the different tissues in the head model, as well as the SpongeStim electrode model (saline-soaked sponge in green and rubber connector in red). The thickness and radius of the different components of the electrode were based on measurements of the actual electrode. **(B)** Global view of the head model used in the optimization pipeline, including representations of the conductive gel underneath the PiStim electrodes. The mask of the left dorsolateral prefrontal cortex (lDLPFC) used in the optimization (BA 46) is also shown in the cortical surface of the head model. **(C,D)** Distribution of the normal component of the *E-field* in the bipolar/multichannel montage (positive/negative values indicate that the E*_n_* field is directed into/out of the cortical surface). The field was divided by its maximum value in each montage (0.52 V/m in the bipolar montage and 0.70 V/m in the multichannel).

The multichannel optimized montage was obtained using the Stimweaver algorithm (Ruffini et al., 2014). This realistic head modeling-based algorithm works under the assumption that the normal component of the *E*-field (E*_n_*) induced by tDCS couples with long pyramidal cells in the cortex, thus leading to cortical excitability changes. The optimal multichannel montage is determined by minimizing the least-square difference between the weighted *E-field* (E*_n_*) induced by the montage and a weighted target map of *E*_n_ ($E_{\text{n}}^{\text{Target}}$). In this optimization, the lDLPFC mask was defined as BA 46 (see Figure 2B) and the weight and *E*_n_ in this area were set to 10 and +0.25 V/m, respectively. The rest of the cortical areas were assigned to a target *E*_n_ of 0 V/m with a low weight (1). The optimization imposed constraints to the maximum current per electrode ($I_{\text{Elec}}^{\text{Max}}$ = 1.0 mA) and total injected current (the sum of all the positive currents $I_{\text{Total}}^{\text{Max}}$ = 2.0 mA).

The optimization was performed in a standard finite element head model of the Colin27 template^3^ following Miranda et al. (2013) and Ruffini et al. (2014; see Figures 2A,B). The head model, shown in Figure 2B, contains representations of the scalp, skull, cerebrospinal fluid (including the ventricles), gray matter, and white matter. For the optimization, models of PiStim electrodes were placed in the scalp in a subset of positions of the international 10-10 EEG system with a radius of 1 cm and a height of 3 mm (Figure 2B), representing the conductive gel beneath them (conductivity of 4.0 S/m). All tissues were represented as homogeneous and isotropic materials with electrical conductivities appropriate to the DC-low frequency range: 0.33 S/m, 0.008 S/m, 1.79 S/m, 0.4 S/m, and 0.15 S/m for the tissues mentioned before, respectively (Miranda et al., 2013).

The *E*-field distribution induced by the bipolar distribution was calculated using the same head model. The electrodes were modeled according to Neuroelectrics’ SpongeStim model: conductive rubber on top of saline soaked sponge. All *E-field* calculations were performed in Comsol (v5.3a)^4^ using its AC/DC package.

The distribution of E*_n_* in the cortical surface in the bipolar and multichannel optimized montages is shown in Figures 2C,D. The multichannel montage distributes the return electrode between the right frontal areas and left temporal cortex, leading to stronger negative E*_n_* values in these regions. In the bipolar montage, this happens in the right frontal area, under the cathode located at Fp2. In terms of fit to the target map, the optimized montage achieved better results, expressed in the cross correlation between the weighted E*_n_* distribution and weighted $E_{\text{n}}^{\text{Target}}$ map: 0.5 and 0.3 for the multichannel and bipolar montages, respectively. The least-square error (ERNI, in units of mV^2^/mm^2^, Ruffini et al., 2014) of the optimized montage is also higher (in absolute value; −6,189 mV^2^/mm^2^) than that of the bipolar montage (−1,285 mV^2^/mm^2^). In terms of average E*_n_* over the lDLPFC surface area, the multichannel montage achieved a higher value (0.07 V/m) than the bipolar montage (0.03 V/m).

### Questionnaire on Tolerability and Participant Blinding of tDCS

Side effects and blinding effectiveness were assessed using a standardized safety questionnaire (Poreisz et al., 2007; Antal et al., 2017). Participants rated the six most common tDCS side effects on a 4-point scale, from 0 = not experienced to 4 = strongly experienced. After each stimulation, the participants gave their opinion as to whether they had received verum or sham stimulation.

### EEG Recording and Preprocessing

We used a 64-channel electrode cap placed over the scalp according to the locations of the international 10-10 standard system with the reference electrode positioned at FCz and at the ground electrode at AFz (EasyCap, Herrsching, Germany). Electrode impedances were always kept below 10 kΩ. EEG was recorded using the BrainVision Recorder Software (Brain Products GmbH, Gilching, Germany). The EEG signal was recorded with a rate of 1,000 S/s and low-pass filtered at 250 Hz.

For EEG data preprocessing, we used Brain Vision Analyzer 2 (Brain Products GmbH, Gilching, Germany). The data was down sampled at 500 S/s, re-referenced to the common average, and filtered (30 Hz low-pass, 0.05 Hz high-pass filter). Semiautomatic raw data inspection and an independent component analysis were applied to remove artifacts. Task-related EEG data was segmented from −1,000 to 1,500 ms poststimulus onset, and continuous resting-state EEG data was divided in 2,000-ms segments. Segmented EEG data was then exported and further analyzed using the FieldTrip toolbox^5^. We performed a time-frequency analysis with a Hanning taper for frequencies from 1 to 30 Hz in steps of 2 Hz in a time window from −500 to 1,000 ms relative to stimulus onset for both tasks, with baseline correction from −500 to 0 ms. Resting-state EEG was fast Fourier transformed with a moving Hanning window in a frequency range from 1 to 30 Hz in steps of 2 Hz and averaged in every subject.

### Statistical Analyses

#### Behavioral Data

Statistical analyses on task accuracy and reaction times (RT) were conducted using the computing environment R [version 3.5.1, R Core Team (2019); R: A Language and Environment for Statistical Computing. R Foundation for Statistical Computing, Vienna, Austria^6^]. For the 2-back task and CPT, accuracy was defined as proportion of correct responses. Due to high ceiling effects in accuracy in the CPT (baseline accuracy: Mean = 99.62%, SD = 0.6%), we restricted our analyses for the CPT to RT only. Behavioral measurements were analyzed using linear mixed-effect models (LME). We assessed normality of behavioral data using the Shapiro–Wilk test and visual inspection of the data (histograms and Q–Q plots). In all models, we included the maximum number of random effects that allowed the model to converge. For 2-back accuracy and RT, our LME included the fixed factors *baseline* (performance at T1), *stimulation* (sham, bipolar, and multichannel stimulation), *time* (during and after stimulation), and all corresponding interactions. Random slopes for *stimulation* and a random intercept were entered as random effects. Because participants completed the CPT only after stimulation and not during stimulation, for CPT RT we fitted an LME including the fixed-effect *stimulation* (sham, bipolar, multichannel) and *baseline* and a random intercept. Degrees of freedom were approximated using the Kenward–Rogers method, analogous to repeated-measure ANOVAs (Kenward and Roger, 1997). In case of significant *F*-values, *post hoc* tests were performed using the Tukey method.

#### Neurophysiological Data

All analyses were performed using significance probability estimations based on Monte-Carlo permutation tests with a cluster-based approach using the FieldTrip toolbox. This nonparametric approach solves the problem of multiple comparisons by cluster correction and avoids assumptions on normally distributed data.

Stimulation effects on task-related and resting-state neurophysiological outcomes were analyzed by one-way repeated-measurement ANOVAs with the within-subject factor *stimulation* (sham, bipolar, multichannel). For task-related time-frequency data and resting-state averaged power spectra, we computed separate ANOVAs for each frequency band (δ: 1–4, θ: 4–8 Hz, α: 8–12 Hz, and β: 12–30 Hz). In case of significant *F*-values, we conducted paired *t*-tests. We decided to use an ANOVA approach for the analysis of neurophysiological data, because a combination of mixed-model and cluster-based analysis is not possible using the FieldTrip toolbox. Although a unified mixed-model analysis would have been preferable in terms of comparability of behavioral and neurophysiological results, we wanted to exploit the advantages of a cluster-based approach for the analysis of physiological data and decided to apply this approach.

Additionally, the interaction of individual baseline-performance and tDCS effects on oscillatory power was examined. Stimulation-induced changes in oscillatory power were computed by subtracting the sham condition from the multichannel and bipolar conditions. This was done for task-related time-frequency representations (TFRs) and resting-state oscillatory power. Pearson correlations between these neurophysiological differences and behavioral baseline performance (2-back accuracy, 2-back RT, and CPT RT) were computed.

## Results

### Tolerability and Blinding of tDCS

Participants were unable to guess better than chance whether they had received active or sham stimulation for all stimulation conditions (sham: $\chi_{\left(1\right)}^{2}$ = 0, *p* = 1.0; bipolar: $\chi_{\left(1\right)}^{2}$ = 0.75, *p* = 0.38; multichannel: $\chi_{\left(1\right)}^{2}$ = 3.0, *p* = 0.08). Neither incidence nor intensity of all side effects differed significantly between stimulation conditions (see Supplementary Table S1).

### tDCS Effects on 2-Back and CPT Performance

Mean accuracy and RT scores are seen in Table 2. Our analyses of 2-back accuracy showed significant main effects of *baseline* (*F*_(1,23.8)_ = 34.83, *p* < 0.001), *stimulation* (*F*_(1,23.8)_ = 6.39, *p* = 0.005), and *time* (*F*_(1,70.9)_ = 9.59, *p* = 0.002). Furthermore, we found a significant interaction of *baseline* × *stimulation* (*F*_(1,23.9)_ = 5.86, *p* = 0.008) and a significant interaction of *baseline* × *time* (*F*_(1,70.9)_ = 9.64, *p* = 0.002), but no significant interaction of *stimulation* × *time* (*F*_(1,70.9)_ = 0.86, *p* = 0.423) or *baseline* × *stimulation* × *time* (*F*_(1,70.9)_ = 0.91, *p* = 0.411).

**Table 2:** Mean (standard deviation) for 2-back and continuous performance task (CPT) accuracy (%) and reaction times (ms) during and after stimulation.

	Time point	Stimulation condition		
		Sham	Multichannel	Bipolar
2-Back accuracy	During	90.68 (5.66)	91.45 (3.82)	91.74 (5.15)
	After	90.67 (6.54)	91.75 (4.89)	91.58 (5.97)
2-Back reaction time	During	509.42 (177.05)	540.61 (185.89)	547.93 (193.46)
	After	496.52 (174.71)	521.69 (201.97)	500.59 (164.33)
CPT accuracy	After	99.63 (0.41)	99.71 (0.36)	99.75 (0.29)
CPT reaction time	After	357.01 (61.34)	362.01 (68.11)	356.12 (67.49)

*Post hoc* tests investigating the *baseline* × *time* interaction revealed a significant lower accuracy slope during stimulation compared to after stimulation (*t*_(77.6)_ = −2.97, *p* = 0.004).

**Figure 3:**
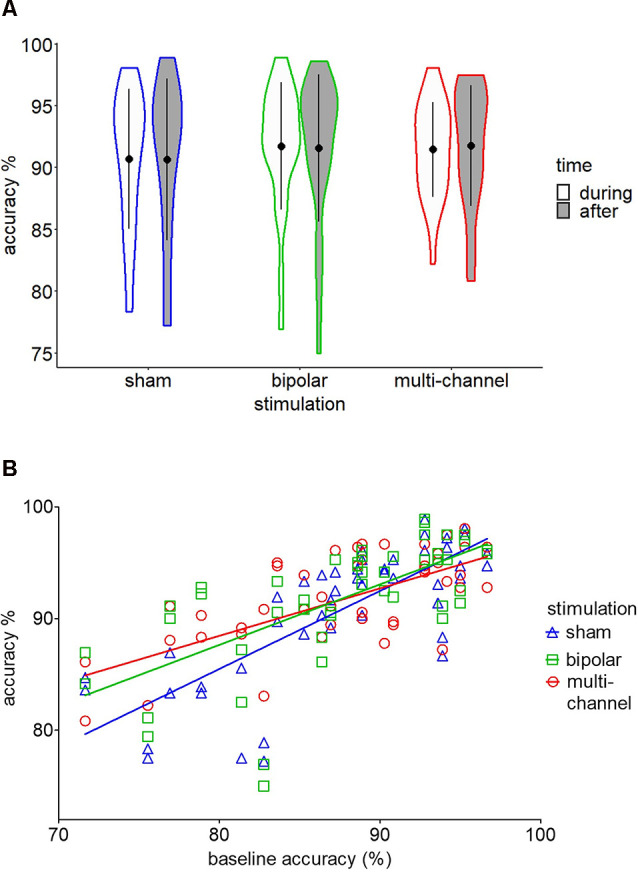
Two-back behavioral results. **(A)** Violin plot for mean (±SD) accuracy scores for sham, bipolar, and multichannel stimulation during and after stimulation. **(B)** Regression lines for multichannel, bipolar, and sham stimulation on baseline accuracy scores.

*Post hoc* tests based on the significant *stimulation* main effect revealed no significant effect for multichannel or bipolar stimulation compared to sham stimulation (all *p* > 0.05; see Figure 3A). *Post hoc* tests following the *baseline* × *stimulation* interaction revealed a significant higher accuracy slope for sham stimulation compared to multichannel stimulation (*t*_(25.9)_ = 3.11, *p* = 0.012; Figure 3B). We found no significant difference in accuracy slopes for sham stimulation compared to bipolar stimulation (*t*_(26.2)_ = 1.95, *p* = 0.114). Importantly, the significant interaction effect of *baseline* × *stimulation* could not be explained by regression to the mean (RTM). To exclude RTM as possible explanation for this interaction, we tested whether the accuracy variances of the multichannel and baseline condition were different (Guilford and Fruchter, 1973; Tu and Gilthorpe, 2007). This test follows the idea that if initially low-performing participants tend to improve under multichannel stimulation and initially high-performing participants do not improve or even decrease in performance, as indicated by Figure 3B, variance for accuracy during multichannel stimulation should be smaller than variance for accuracy at baseline measurement. This was found to be true, as multichannel accuracy had a significant lower variance than baseline accuracy (*t*_(22)_ = 3.49, *p* < 0.01). Also, multichannel accuracy variance was significantly lower than sham accuracy variance (*t*_(22)_ = 2.59, *p* < 0.02; see Figure 3A). Still, this result could reflect a training effect, with a more homogenous accuracy through repetition of the 2-back task. In this case, variances for bipolar and sham stimulation should also be significantly decreased compared to baseline variance. This assumption was not confirmed, as accuracy variances were not decreased under bipolar (*t*_(22)_ = 1.69, *p* > 0.05) or sham (*t*_(22)_ = 1.34, *p* > 0.05) stimulation compared to baseline.

Our mixed models performed for 2-back RT and CPT RT revealed a significant main effect for *baseline* (2-back RT:* F*_(1,24.3)_ = 36.04, *p* < 0.001; CPT RT: *F*_(1,24)_ = 143.49, *p* < 0.001); all other main effects and interactions were not significant (all *p* > 0.05). Therefore, no subgroup analyses were performed.

### tDCS Effects on Neuronal Oscillations

**Figure 4:**
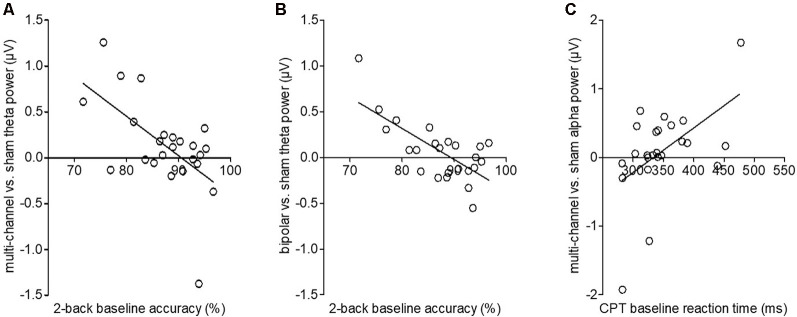
Stimulation-induced changes in theta and alpha power averaged over time depending on behavioral baseline performance. **(A)** Correlation of multichannel vs. sham theta power with individual 2-back baseline accuracy. The y-axis depicts the difference of mean theta power for multichannel–sham stimulation. The regression line shows a decrease in stimulation-induced theta power with increasing baseline accuracy. **(B)** Correlation of bipolar vs. sham theta power with individual 2-back baseline accuracy. The y-axis depicts the difference of mean theta power for bipolar–sham stimulation. The regression line shows a decrease in stimulation-induced theta power with increasing baseline accuracy. **(C)** Correlation of multichannel vs. sham alpha power with individual CPT baseline reaction times (RT). The y-axis depicts the difference of mean alpha power for multichannel–sham stimulation. The regression line shows an increase in stimulation-induced alpha power with increasing baseline accuracy.

**Figure 5:**
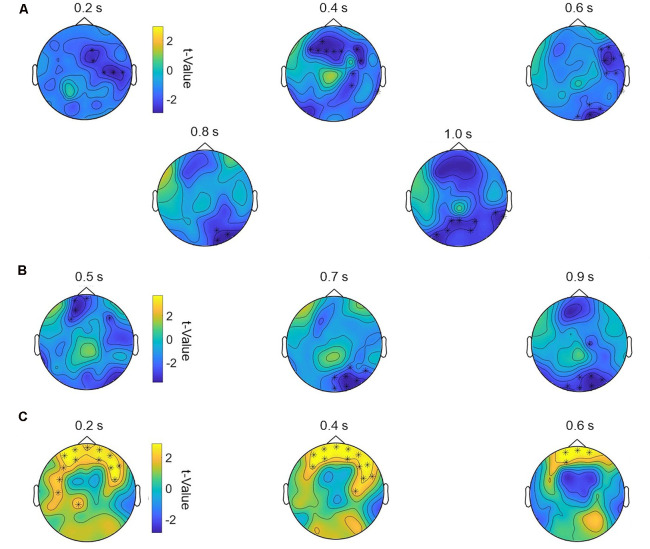
Topography of the significant correlations for theta and alpha power following multichannel and bipolar stimulation compared to sham stimulation. **(A)** Correlation of multichannel vs. sham theta power with individual 2-back baseline accuracy from 200 to 1,000 ms in steps of 200 ms. Significant channels: F3, F4, C4, P4, O2, F8, T8, P8, Fz, Pz, FC2, FC6, FT10, TP10, F1, F2, P1, P2, AF3, AF4, CP4, PO3, PO4, F5, F6, C6, FT8, TP8, PO7, PO8, POz, Oz, FCz. **(B)** Correlation of bipolar vs. sham theta power with individual 2-back baseline accuracy from 500 to 1,000 ms in steps of 200 ms. Significant channels: Fp1, F3, P4, O1, O2, T8, P7, P8, Fz, FC6, CP6, F1, C2, AF3, FC3, CP4, PO4, F6, C5, P6, PO7, PO8, Fpz, POz. **(C)** Correlation of multichannel vs. sham alpha power with individual CPT baseline RT from 200 to 600 ms in steps of 200 ms. Significant channels: Fp1, Fp2, F3, C4, F8, CP1, FC5, FC6, AF3, AF4, F5, F6, C5, AF7, AF8, FT7, FT8, Fpz.

One-way repeated-measurement ANOVAs on event-related oscillations revealed no main effect of *stimulation* for all frequency bands in the 2-back task. Correlation analyses showed no significant correlations between 2-back RT and oscillations (all *p* > 0.05). However, we found significant negative correlations between behavioral 2-back baseline accuracy and stimulation-induced increase in power in the theta band for multichannel (*p* = 0.009) and bipolar (*p* = 0.008) stimulation compared to sham. The worse the participant initially performed, the more theta power was detected after multichannel and bipolar stimulation compared to sham stimulation (Figures 4A,B). For multichannel stimulation, this effect was seen in frontal and occipital areas from 200 to 1,000 ms poststimulus (Figure 5A). For bipolar stimulation, we observed this effect in frontal and occipital areas from 500 to 900 ms poststimulus (Figure 5B).

For CPT event-related oscillations, we found no main effect of *stimulation*, but a significant positive correlation between behavioral baseline CPT RT and stimulation induced changes in alpha power for multichannel compared to sham stimulation (*p* = 0.01). Subjects with higher baseline RT showed higher alpha power after multichannel compared to sham stimulation (Figure 4C). This effect occurred in a frontal area from 200 to 600 ms poststimulus onset (Figure 5C).

Analyses on resting-state oscillations revealed no significant effects for *stimulation* or significant correlations for behavioral and oscillatory activity.

## Discussion

Here we compared the effects of two different tDCS montages targeting the lDLPFC, taking into account the influence of individual baseline performance. Bipolar and multichannel stimulations were both well tolerated and effectively blinded. We found no effect of stimulation on behavioral performance or neuronal oscillations comparing the classical bipolar or the multichannel montage with sham stimulation. However, we observed an interaction of stimulation and baseline performance for behavioral and neurophysiological outcomes. Multichannel stimulation influenced WM performance depending on the baseline performance level, leading to decreased variability in accuracy between subjects. Initially low-performing participants tended to improve their WM performance while initially high-performing participants tended to worsen their performance compared to sham stimulation. Furthermore, changes in neuronal oscillations following tDCS correlated with behavioral baseline performance. The worse the participant initially performed, the more the WM task-related theta power was increased following multichannel and bipolar stimulation compared to sham stimulation. Interestingly, alpha power in the nontarget task was also influenced by multichannel stimulation depending on initial baseline performance.

In line with previous studies, we partly show that multichannel stimulation might lead to pronounced tDCS effects when compared to sham stimulation. However, we were not able to show a superiority of multichannel stimulation compared to bipolar stimulation as it was demonstrated in the resting-state motor network (Fischer et al., 2017). Using the sham stimulation as a reference, multichannel montage produced stronger effects on behavioral and neurophysiological outcomes than the bipolar montage. Comparing multichannel and bipolar effects directly, no superiority can be seen for neither of the two montages. A possible explanation could be that enhanced effects of tDCS on the motor network cannot generalize to other brain areas. Additionally, it could be argued that resting-state and task-specific active networks are affected differently by tDCS. According to the network model, the interaction of different factors has an influence on the stimulation effects. With online brain activity, unlike offline brain activity in resting-state networks, different factors influence the effects of stimulation such as state of activation, task difficulty, level of performance, and cognitive functions or strategies involved (Li et al., 2015; Fertonani and Miniussi, 2017). Also, it is important to note that WM is a complex, high-level cognitive function, composed of different subprocesses (Baddeley, 2003).

The lack of significant effects comparing multichannel and bipolar stimulation with sham stimulation for behavioral and neurophysiological outcomes could be due to interindividual factors, which vary the responses to tDCS. Recent meta-analyses report minor or even negative effects of tDCS on WM performance (Brunoni and Vanderhasselt, 2014; Dedoncker et al., 2016; Hill et al., 2016; Mancuso et al., 2016), stressing the need to investigate factors potentially influencing tDCS outcome, such as anatomical features, baseline neurophysiological state, or development and aging (Horvath et al., 2014; Krause and Cohen Kadosh, 2014; Moliadze et al., 2015, 2018; Filmer et al., 2019; for review; see Li et al., 2015). Accordingly, when including participants’ individual baseline performance in our analyses, a significant interaction between stimulation and baseline performance can be observed. Importantly, this effect was not due to RTM as tests on variances show significant decreased accuracy variances following multichannel stimulation, but not following bipolar or sham stimulation, compared to baseline accuracy variance. Following Krause et al. (2013), it can be assumed that there is an optimal level of prefrontal activation based on an excitation/inhibition (E/I) balance, measured by glutamate/GABA concentration (Stagg et al., 2009; Clark et al., 2011). Based on this theory, tDCS can lead to reinstatement of an optimal E/I balance but can also lead to overactivation and worsening of performance. This might be the reason for improved WM performance in initially low performers and worse performance in initially high performers. This interaction is in line with previous studies reporting an increase for low-performing and a decrease for high-performing participants for different WM parameters (London and Slagter, 2015; Gözenman and Berryhill, 2016).

Our results for task-related oscillations correspond to previous studies reporting increased theta and alpha oscillations following lDLPFC stimulation (Zaehle et al., 2011; Boonstra et al., 2016; Jones et al., 2017). Theta activity has been shown to be crucial for WM processes (Gevins et al., 1997; Klimesch et al., 2005; Pesonen et al., 2007; Lisman, 2010), memory maintenance (Jensen and Tesche, 2002), and retrieval (Klimesch et al., 2001). The stimulation-induced change in theta power we have observed, depending on initially baseline performance, may therefore indicate increased cognitive processing for initially low-performing participants. Our nontarget task, the CPT, investigated response inhibition and attention (Rosvold et al., 1956). Alpha oscillations following stimulus onset have been increased after multichannel stimulation depending on initially baseline performance. Stimulation-induced changes of alpha oscillations during this nontarget task suggests a transfer effect of the stimulation to functions that have not been entrained during stimulation (Allenby et al., 2018). The increase in alpha following stimulus onset could reflect increased response inhibition through the inhibition of related cortical areas (Klimesch, 1996; Schmiedt-Fehr et al., 2009).

In contrast to Zaehle et al. (2011), changes in oscillatory power were not associated with alterations in WM performance after stimulation. A reason for missing performance changes might be that effects of stimulation on WM performance tend to be relatively small (Hill et al., 2016; Mancuso et al., 2016). Neurophysiological activity is potentially more sensitive to stimulation than behavioral performance. The neurophysiological effects therefore suggest that tDCS together with the 2-back WM task has activated the underlying neurophysiological network beyond the duration of stimulation but not to a sufficient extent to lead to effects at the behavioral level. Following this idea, both stimulation and task engagement led to neurophysiological changes. This could also explain the missing stimulation effects on resting-state oscillations. In line with previous studies, we did not observe effects on resting-state oscillatory power following stimulation (Horvath et al., 2015; Gordon et al., 2018; Hill et al., 2019). Our results suggest, that effects on neurophysiological outcomes are only detectable during network activation through task performance, representing a state-dependency of stimulation effects (Silvanto et al., 2007; Learmonth et al., 2015; for review see Hsu et al., 2016).

Multichannel stimulation, in combination with the initial behavioral baseline performance, led to effects on both behavioral and neurophysiological outcomes, while bipolar stimulation only affected oscillations in the target WM task. While the maximum injected current was the same across active montages, the observed differences may have arisen due to the higher total current used in the multichannel compared to the bipolar montage, thus leading to a higher average E*_n_* field in the lDLPFC. Increasing the current in the bipolar montage would increase the average E*_n_* field in the same proportion. A previous study seems to point to a relationship between current density in the lDLPFC and improvement in WM performance (Kim et al., 2014). This study, however, has some technical limitations, especially in the electrical conductivities assigned to the tissues represented in the models. Another potential factor that can affect the results is the focality of the E*_n_* field distribution, which is much higher in the optimized montage than in the bipolar montage, especially in the area outside the target. Lack of focality of the bipolar montage introduces confounding factors when analyzing the data, as stimulation of other cortical areas might affect performance of the subjects in these tasks. It should be noted here that although it is possible to use multichannel montages that achieve greater focality of stimulation (like the 4 × 1 montage with one central anode), this comes at the cost of a lower-average E*_n_* field on the target area, which may reduce the effects of stimulation. The used multichannel approach strikes a balance between focality and average E*_n_* on target.

Additionally, the multichannel and bipolar montage used different electrodes, which may have provided sensory cues to the subjects as to the method of stimulation. Thus, no effective blinding of the applied montage at each visit could take place. Therefore, we cannot exclude that the subjects had implicit assumptions about the effectiveness of the different montages, which in turn may have had an impact on the measured outcomes. However, for both montages we achieved effective blinding in terms of verum or sham stimulation and all subjects were naive to stimulation and the study aim of comparing the effectiveness of montages.

Another limitation of this study is related to the lack of subject-specific personalized head models. These are important, as interindividual anatomical features such as skull thickness and cortex folding have been shown to have large influence on tDCS current flow (Opitz et al., 2015; Laakso et al., 2016). These personalized models would provide means to calculate the average E*_n_* in the lDLPFC for each subject, which could then be used as an additional term in the statistical analysis of the data, as performed by Laakso et al. (2019). Also, the influence of baseline performance could have been investigated in more details if a larger sample size would have been collected. This would have allowed us to study the effects of stimulation in different subgroups.

In summary, and considering the limitations we have highlighted, our results demonstrate the importance of taking into account interindividual baseline performance and montage when stimulating the lDLPFC. Several studies have shown a limited effectiveness of tDCS on WM, often expressed in a low response rate. Therefore, our study helps to identify the factors that determine whether a subject benefits from stimulation. Moreover, sharing partly “null results” (1) will have a positive impact on future research questions and (2) will improve knowledge acquisition of noninvasive transcranial brain stimulation techniques.

## Data Availability Statement

Due to ethical restriction, the data from this study will not be able to be accessible from public domain. The data are available from the corresponding author upon request. Requests to access these datasets should be directed to splittgerber@med-psych.uni-kiel.de.

## Ethics Statement

The studies involving human participants were reviewed and approved by Ethics Committee of the Faculty of Medicine Kiel University, Kiel. The patients/participants provided their written informed consent to participate in this study.

## Author Contributions

MSp, MSi, and VM designed the experiment. MSp administered the experiment, collected, and analyzed the data. RS, RN, and GR performed modeling. MSp, RS, MSi, and VM wrote the manuscript. MSp, HB, CB-Z, AP-K, and KK developed the tasks for the study. All authors discussed the results and implications and commented on the manuscript at all stages. All authors contributed to the article and approved the submitted version.

## Conflict of Interest

RS and RN are employees at Neuroelectrics. GR is a co-founder of Neuroelectrics, a company that manufactures tDCS and EEG technology.

The remaining authors declare that the research was conducted in the absence of any commercial or financial relationships that could be construed as a potential conflict of interest.

## Footnotes

^1^http://www.neurobs.com

^2^http://faculty.sites.uci.edu/starklab/mnemonic-similarity-task-mst

^3^http://www.bic.mni.mcgill.ca/ServicesAtlases/Colin27

^4^http://www.comsol.com

^5^http://fieldtrip.fcdonders.nl/

^6^https://www.R-project.org/
