# Supplementary material for: Individual Baseline Performance and Electrode Montage Impact on the Effects of Anodal tDCS Over the Left Dorsolateral Prefrontal Cortex
Source: Front Hum Neurosci. 2020 Sep 8;14:349. doi: 10.3389/fnhum.2020.00349 (PMC7506510; doi:10.3389/fnhum.2020.00349)
Supplement: Supplementary file 1 [file Table_1.DOCX]

Individual baseline performance and electrode montage impact on the effects of anodal tDCS over the left dorsolateral prefrontal cortex

Supplementary Material

# Supplementary Table

**Table S1** Incidence (in %) and intensity (scale 1–5, Ø) of side effects for multi-channel, bipolar and sham stimulation.

|  |  | multi vs. bipolar | multi vs. sham | bipolar vs. sham |
| --- | --- | --- | --- | --- |
| Itching sensation | Incidence  Intensity | 79.2/75  1.57/1.42 | 79.2/65.2  1.57/1.33 | 75/65.2  1.42/1.33 |
| Pain | Incidence  Intensity | 16.7/16.7  1.25/1.25 | 16.7/21.7  1.25/1 | 16.7/21.7  1.25/1 |
| Burning sensation | Incidence  Intensity | 58.3/45.8  1.36/1.5 | 58.3/52.2  1.36/1.11 | 45.8/52.2  1.5/1.11 |
| Warmth/Heat | Incidence  Intensity | 20.8/29.2  1/1.5 | 20.8/30.4  1/1 | 33.3/30.4  1.5/1 |
| Metallic/Iron taste | Incidence  Intensity | 0/0  n.a. | 0/0  n.a. | 0/0  n.a. |
| Fatigue/Decreased alertness | Incidence  Intensity | 33.3/25  1.5/1.2 | 33.3/30.4  1.5/1.67 | 27.8/30.4  1.2/1.67 |

*n.a. = not applicable.*
